# Bisphenol A in Oocytes Leads to Growth Suppression and Altered Stress Performance in Juvenile Rainbow Trout

**DOI:** 10.1371/journal.pone.0010741

**Published:** 2010-05-20

**Authors:** Neelakanteswar Aluru, John F. Leatherland, Mathilakath M. Vijayan

**Affiliations:** 1 Department of Biology, University of Waterloo, Waterloo, Ontario, Canada; 2 Department of Biomedical Sciences, Ontario Veterinary College, University of Guelph, Guelph, Ontario, Canada; University of Lethbridge, Canada

## Abstract

**Background:**

Bisphenol A (BPA), used in the manufacture of plastics, is ubiquitously distributed in the aquatic environment. However, the effect of maternal transfer of these xenobiotics on embryonic development and growth is poorly understood in fish. We tested the hypothesis that BPA in eggs, mimicking maternal transfer, impact development, growth and stress performance in juveniles of rainbow trout (*Oncorhynchus mykiss*).

**Methodology/Principal Findings:**

Trout oocytes were exposed to 0, 30 and 100 µg.mL^−1^ BPA for 3 h in ovarian fluid, followed by fertilization. The embryos were maintained in clean water and sampled temporally over 156-days post-fertilization (dpf), and juveniles were sampled at 400-dpf. The egg BPA levels declined steadily after exposure and were undetectable after 21- dpf. Oocyte exposure to BPA led to a delay in hatching and yolk absorption and a consistently lower body mass over 152-dpf. The growth impairment, especially in the high BPA group, correlated with higher growth hormone (GH) content and lower GH receptors gene expression. Also, mRNA abundances of insulin-like growth factors (IGF-1 and IGF-2) and their receptors were suppressed in the BPA treated groups. The juvenile fish grown from the BPA-enriched eggs had lower body mass and showed perturbations in plasma cortisol and glucose response to an acute stressor.

**Conclusion:**

BPA accumulation in eggs, prior to fertilization, leads to hatching delays, growth suppression and altered stress response in juvenile trout. The somatotropic axis appears to be a key target for BPA impact during early embryogenesis, leading to long term growth and stress performance defects in fish.

## Introduction

Bisphenol A is a common plasticizer used in the production of polycarbonate and epoxy resins and is widely distributed in the aquatic environment [1]. This chemical is toxic to fish at environmentally relevant levels, and chronic exposures leads to embryonic deformities and abnormal development in early life stages [2], [3], as well as growth retardation and reproductive impairment in adults [4], [5]. As with mammals, this chemical mimics the action of female sex steroid hormone 17β-estradiol (E2) in fish, including the synthesis of egg yolk protein vitellogenin [6]. Indeed the majority of studies on BPA exposure in fish have focused on its role as a xenoestrogen [6], whereas the action of this chemical on other endocrine systems has received scant attention [7]. In mammals, BPA disrupts the GH/IGF (somatotropic) axis and the action of thyroid hormones leading to abnormalities in growth and development [8]. Majority of those studies utilized *in vitro* cell systems to understand the mechanism of action of BPA in impacting the functioning of the somatotropic axis, while demonstration of its disruption by BPA in whole animal models are lacking.

In fish, as in mammals, the endocrine regulation of growth and development is under the control of the somatotropic axis, including GH, IGF-1 and IGF-2, their receptors and plasma binding proteins [9]. While recent studies suggest that xenobiotics may target this key axis regulating growth in fish especially during the early life stages [7], a role for BPA in this developmental and growth abnormalities are unclear. This is a cause for concern given the recent findings that BPA accumulates in lipid depots, which could potentially lead to maternal transfer of this chemical. Indeed large scale accumulation of xenobiotics occurs in eggs *via* maternal transfer in feral population from polluted waters [10], [11], [12], while the longer-term implications of this on development and growth in fish are currently unknown.

We tested the hypothesis that maternal transfer of BPA into eggs, prior to fertilization, leads to developmental defects and long-term impact on growth and performance in fish. To test this, we exposed rainbow trout (O*ncorhynchus mykiss)* oocytes to different concentrations of BPA for 3 h just prior to fertilization, mimicking accumulation by maternal transfer, and monitored development, growth and stress performance over a 1 year period. Temporal changes in GH levels and mRNA abundances of GH, IGF as well as their receptors were determined to assess whether the somatotropic axis was a possible target for BPA impact. The longer-term impact on fish performance was assessed by subjecting the juveniles to a standardized stressor challenge and measuring plasma cortisol and glucose responses [13]. Plasma cortisol and glucose elevations in response to an acute stressor exposure are an evolutionarily conserved adaptive response to regain homeostasis in vertebrates, and a well-established marker of stress performance in animals [14], [15].

## Results

### Bisphenol A (BPA) concentrations and VTG expression

**Figure 1:**
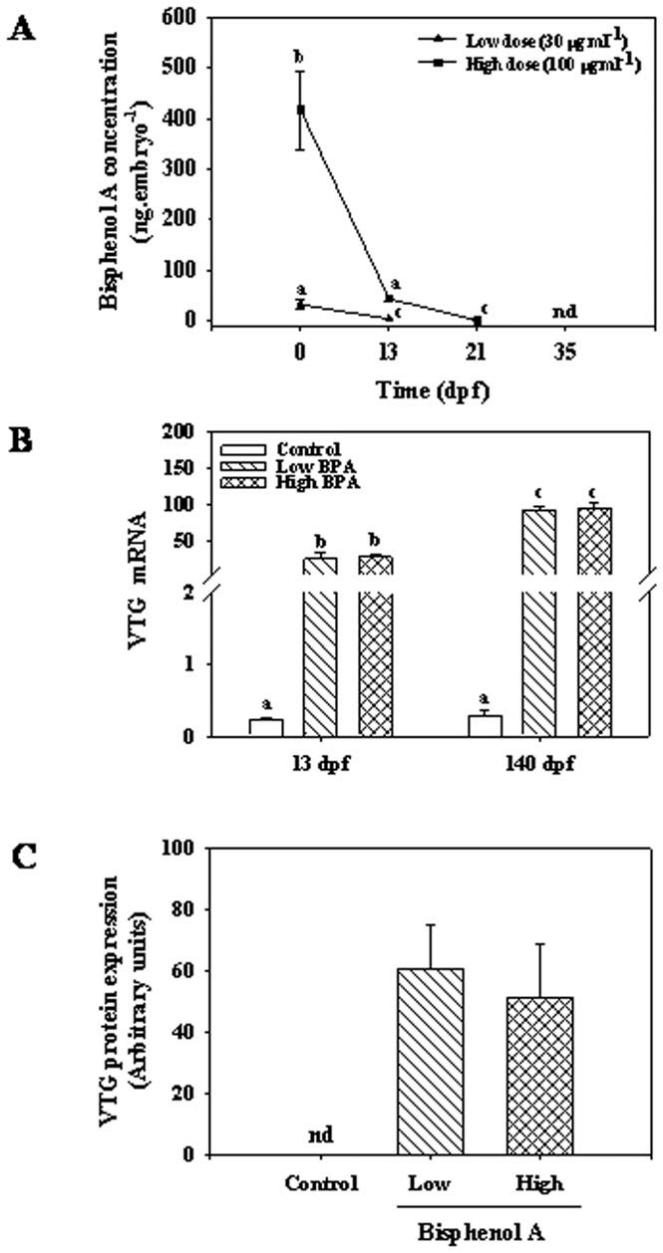
Bisphenol A (BPA) content and vitellogenin (VTG) expression in trout embryos. Temporal changes in BPA concentrations (A) and VTG mRNA abundance (B) and protein expression (C) in rainbow trout embryos after 3 h acute exposure of oocytes to low (30 µg. ml^−1^) and high (100 µg.ml^−1^) dose of BPA. BPA was dissolved in ethanol and added to the ovarian fluid and after 3 h exposure oocytes were fertilized with untreated sperm, water hardened and maintained at 8.5°C. BPA was quantified in embryos collected soon after exposure (3 h) and at 13-, 21- and 35-dpf using LC-MS/MS method. The minimum detection limit of BPA was 75–80 ng.g^−1^ wet weight of tissue (n = 3; each replicate is a pool of 6 embryos). nd denotes non-detectable levels of BPA. VTG mRNA levels in embryos at 13- and 140-dpf (B) were determined by quantitative real-time PCR (qPCR). Liver VTG protein expression (C) was determined in the juveniles at 140-dpf by Western blotting. VTG protein content was detected using anti-trout VTG antibody raised in rabbit. VTG protein was non-detectable (nd) in the control group; values represent mean + SEM (n = 6–8 fish); bars with different letters are statistically significant (ANOVA, p<0.05).

BPA was undetectable in the control eggs and embryos. BPA concentrations after 3 h exposure were 32±9 and 417±78 ng.oocyte^−1^, which was 1.2% and 4.5% of the toxicant in the ovarian fluid in the low and high BPA treatment groups, respectively (Fig. 1A). The BPA content in the embryos dropped by at least 90% in the low (2.4±0.7 ng.embryo^−1^) and high (44±5 ng.embryo^−1^) groups at 13-dpf (Fig. 1A). By day 21, the BPA levels were below the level of detection in the low group, whereas it had dropped further to 2.6±0.1 ng.embryo^−1^ in the high group (Fig. 1A). BPA levels were below detection in both groups at 35-dpf and onwards.

As BPA is estrogenic in fish, VTG gene expression was used to confirm BPA exposure. There was a significant elevation in VTG mRNA abundance in the embryos reared from the BPA exposed oocytes compared to control at 13-dpf (when BPA was present in the embryos) and 140-dpf (when BPA was undetectable; Fig. 1B). No significant differences in VTG mRNA levels were observed between low and high BPA groups either at 13-dpf or 140-dpf (Fig. 1B). Liver VTG protein expression was also significantly higher in fish (140 dpf) reared from BPA exposed oocytes compared to the control group (Fig. 1C). No significant difference in VTG protein expression was observed between low and high BPA groups (Fig. 1C). Gonadal histology at 150-dpf showed no significant sex differences in the BPA groups compared to the control (data not shown).

### Phenotypic changes, survival and growth

**Figure 2:**
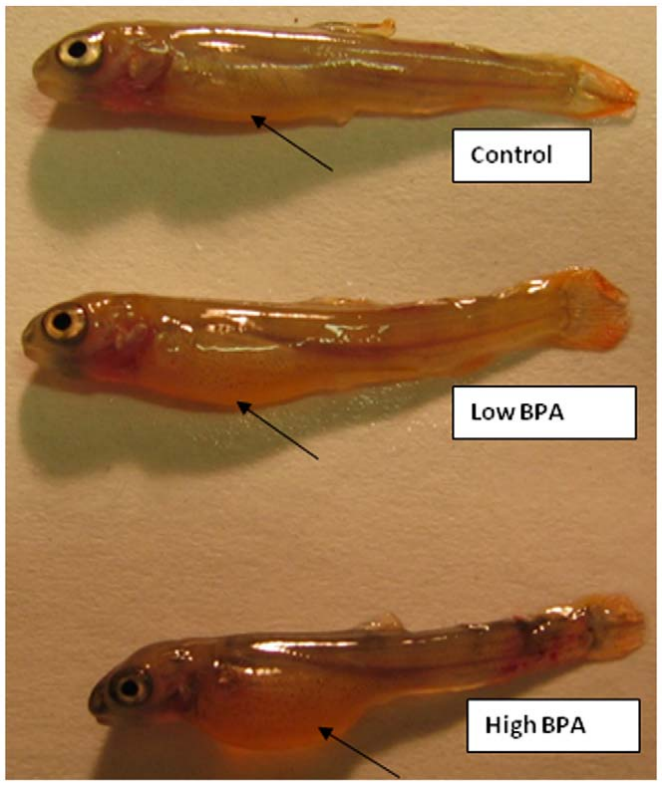
Impact of bisphenol A (BPA) on embryo development. Phenotypic changes post-hatch in trout embryos (65 dpf) treated with BPA. Rainbow trout oocytes were acutely exposed to either low (30 µg. ml^−1^) or high (100 µg.ml^−1^) BPA for 3 h, fertilized with untreated sperm, water hardened and maintained at 8.5°C. Image clearly shows morphological differences, including smaller size and the presence of yolk (arrow shown) in the BPA groups compared to the control.

**Figure 3:**
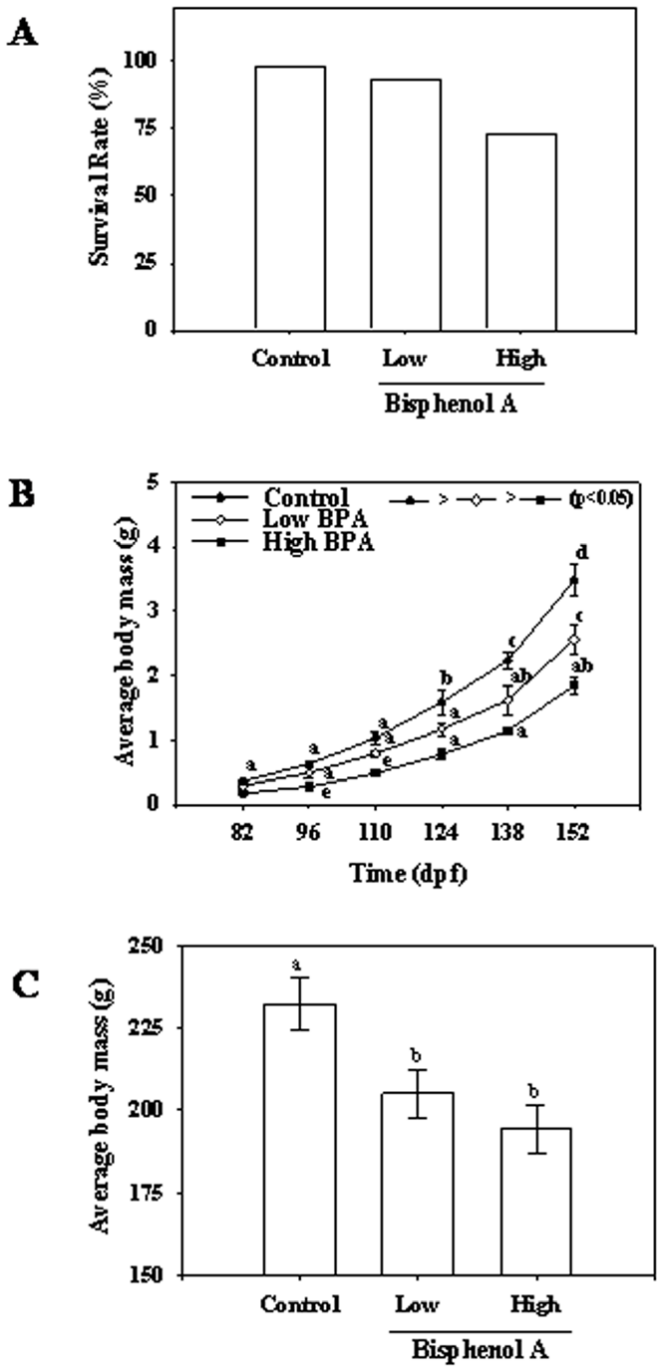
Impact of bisphenol A (BPA) on survival and growth. (A) Percent survival of trout embryos was calculated at the end of the experimental period (400-dpf). (B) Temporal changes in average body mass (g), measured every two weeks after the time of first feed (65 dpf), in the control and BPA exposed groups during development, and (C) in juveniles at 400 dpf. Oocytes were exposed to either control (vehicle alone) or BPA at 30 µg.ml^−1^ (low) or 100 µg.ml^−1^ (high) for 3 h and fertilized with untreated sperm. Fertilized eggs were incubated at 8.5°C and were sampled at various time points during development; values represent mean + SEM (n = 6); bars with different letters are statistically significant (two-way ANOVA for temporal changes and one-way ANOVA for single time point; p<0.05).

BPA exposure did not affect fertilization rate of the eggs. However, embryonic development was delayed in the high BPA group compared to the control and low BPA exposed groups. In the BPA exposed group, there was a delay in hatching, yolk reabsorption and first feeding by about 7 days compared to other two groups. This was more pronounced in the high BPA group compared to the low BPA group (Fig. 2). There was no mortality observed in any of the treatments up until hatching. Between hatching and first feeding, 10% and 30% mortality rates were observed in the low and high BPA groups, respectively (Fig. 3A). There after, no mortalities were observed in any of the treatments. The body mass was significantly lower in the high dose group at all the time points compared to the control group (Fig. 3B). At the end of the experiment (400-dpf), the mean body masses of both high and low BPA treatment groups were significantly lower than that of the control group (Fig. 3C).

### Growth hormone (GH)

**Figure 4:**
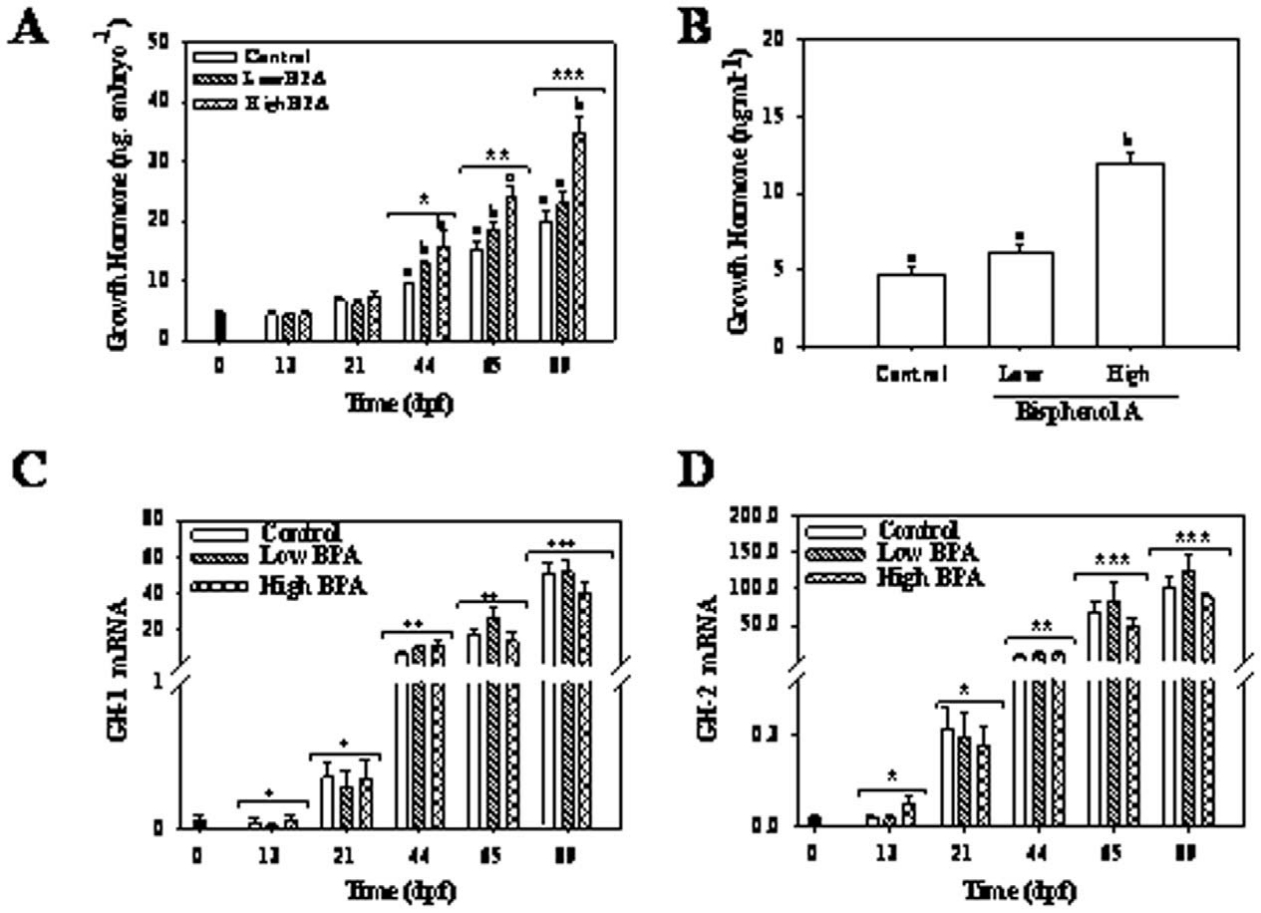
Impact of bisphenol A (BPA) on growth hormone (GH) content and gene expressions. Temporal changes in GH content in the control and BPA treated groups during early development (A) and plasma GH levels at 400-dpf (B); Temporal changes in GH1 (C) and GH2 (D) mRNA abundances in trout exposed to control and two different concentrations of BPA. Maternal transcript levels were measured in freshly fertilized eggs and represented as 0-dpf (dark colored bar). GH was determined using trout specific anti-GH antibody as described in the materials and methods. Two-way ANOVA was used to determine the effect of time, treatment and interaction effects on GH levels and transcript levels during development (Bonferonni posthoc test; p<0.05). Different letters represent differences between treatments at each time point. Asterisk (*) represent effect of time on GH content. One way ANOVA was used to determine the effect of BPA on plasma GH levels. All values represent mean + SEM (n = 7).

Whole embryo GH levels showed no significant differences between different treatments at time 0, 13- and 21-dpf (Fig. 4A). At 44-dpf, there was a significant difference in embryo GH levels between the control and the BPA treatment groups, with significantly elevated GH levels in the treated groups compared to the controls; there was no significant difference in the levels observed between the two BPA treatment groups (Fig. 4A). At 65-dpf, there was a significant dose-dependent increase in embryo GH content, whereas in the 89-dpf juveniles, the values for the animals in the higher BPA treatment group had significantly higher GH levels compared to those of the lower BPA treatment group and the controls (Fig. 4A). This difference was also maintained at 400-dpf, where plasma GH levels in the high BPA group were significantly elevated compared to the low BPA and control groups (Fig. 4B).

**Figure 5:**
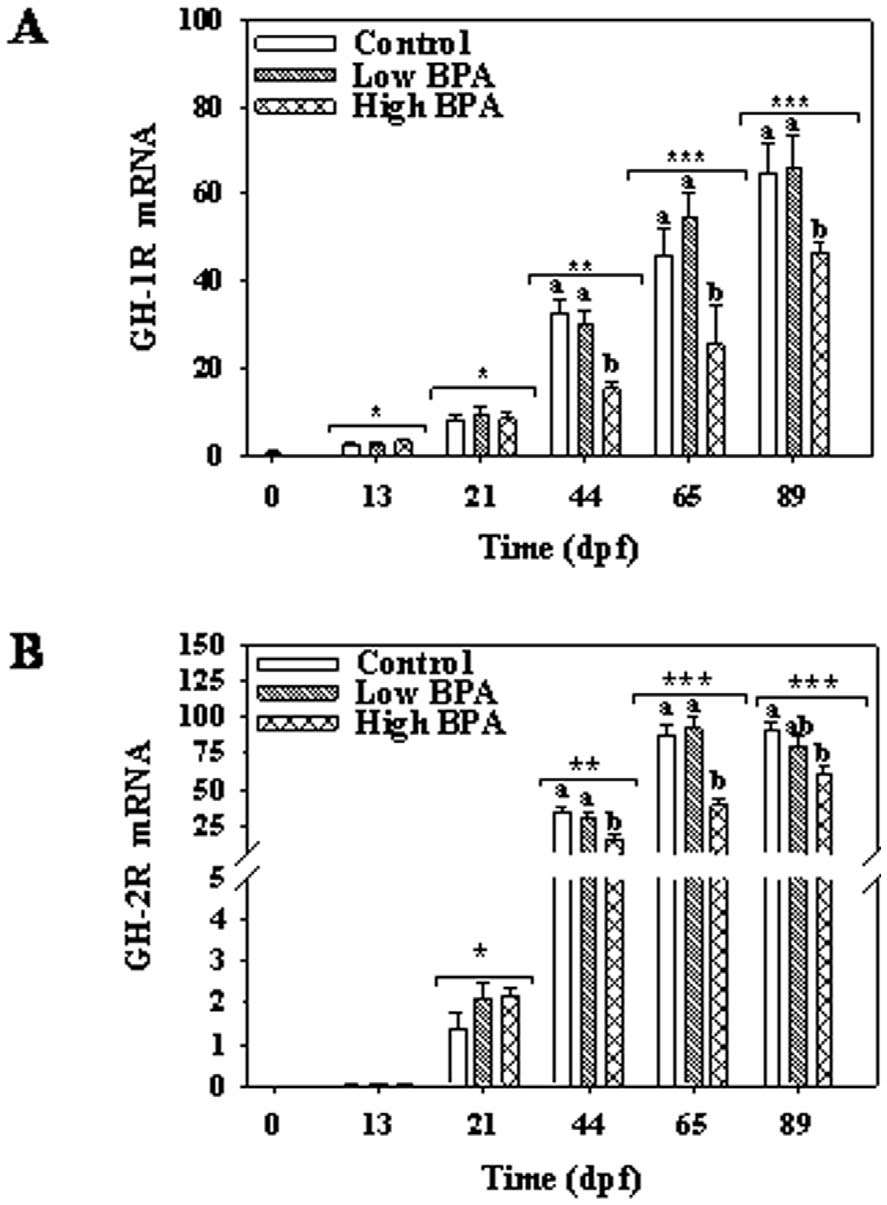
Impact of bisphenol A (BPA) on growth hormone (GH) receptors gene expressions. Temporal changes in GH-1 receptor (GH-1R; A) and -2 receptor (GH-2R; B) mRNA abundances during rainbow trout development. See Fig. 2 legend for experimental details. Maternal transcript levels were measured in freshly fertilized eggs and represented as 0 dpf (dark colored bar). Two-way ANOVA was used to determine the effect of time, treatment and interaction effects on the transcript levels during development (Bonferonni posthoc test; p<0.05). Different letters represent differences between treatments at each time point. Asterisk (*) represent effect of time on the transcript levels. All values represent mean + SEM (n = 7 fish).

GH-1 and GH-2 mRNA abundances also showed a temporal increase during development (Figs. 4C and 4D). No effect of BPA on GH transcript levels was observed at any of the time points; however, GH-receptor transcripts were impacted by the high BPA treatment (Fig. 5A and 5B). Both GH-1 (Fig. 5A) and GH-2 receptor (Fig. 5B) levels were lower in the higher BPA treatment group at 44-, 65- and 89-dpf compared to control and low BPA group.

### Insulin-like growth factors (IGFs)

**Figure 6:**
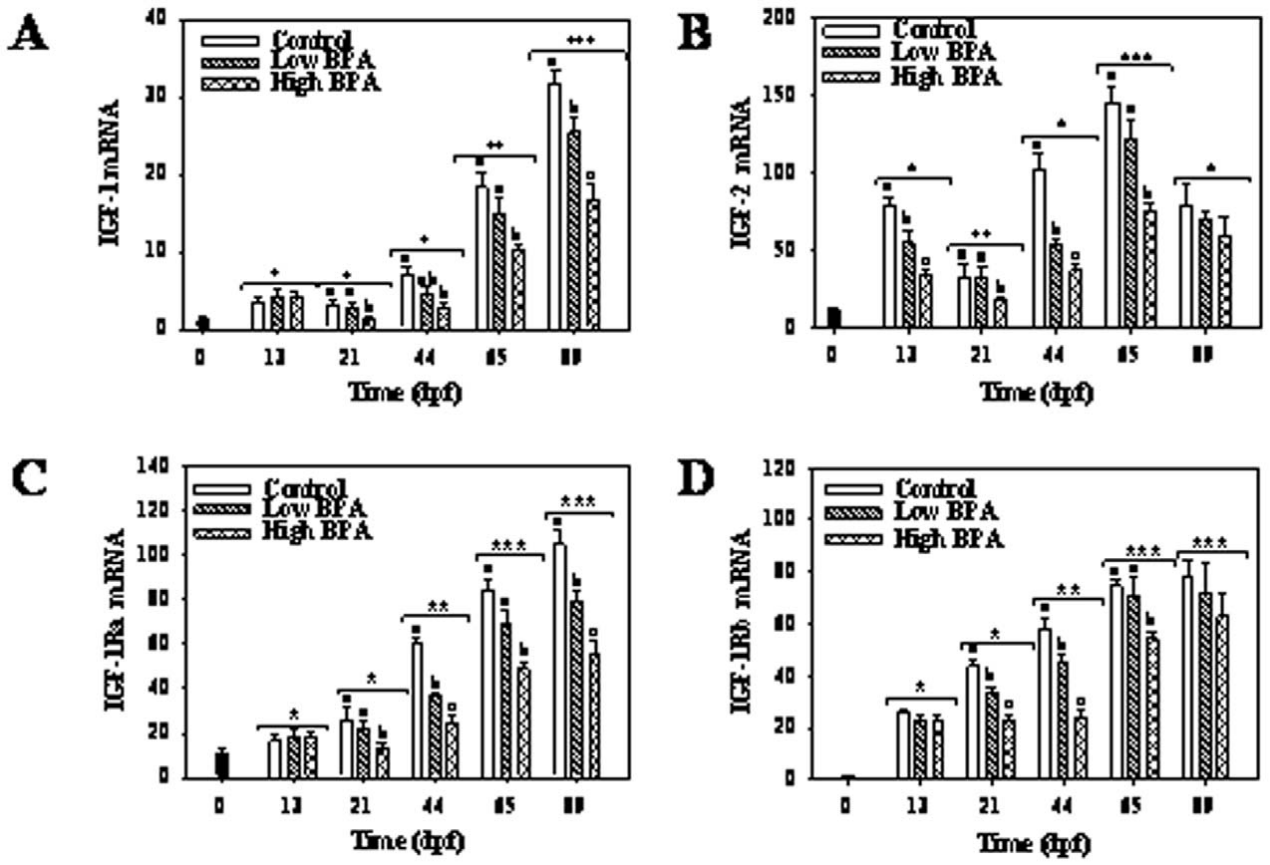
Impact of bisphenol A (BPA) on insulin-like growth factors (IGFs) and their receptors gene expressions. Temporal changes in IGF-1 (A) and IGF-2 (B) and IGF-1 receptor a (IGF-1 Ra; C) and b (IGF-I Rb; D) mRNA abundances in rainbow trout embryos in response to BPA treatment. See the Fig. 2 legend for experimental details. Maternal transcript levels were measured in freshly fertilized eggs and represented as 0-dpf (dark colored bar). Two-way ANOVA was used to determine the effect of time, treatment and interaction effects on the transcript levels during development (Bonferonni post hoc test; p<0.05). Different letters represent differences between treatments at each time point. Asterisk (*) represent effect of time on mRNA abundances. All values represent mean + SEM (n = 7).

There was a time-dependent increase in the transcript levels of both IGF-1 and IGF-2 mRNA throughout development (Fig. 6A and B). In the higher BPA treatment group IGF-1 mRNA levels were lower than controls at all time points except at 13-dpf, whereas in the lower BPA treatment group significantly lower IGF-1 mRNA levels relative to the controls, were only apparent at 89-dpf (Fig. 6A).

IGF-2 mRNA levels were significantly higher than IGF-1 levels at all developmental stages (Fig. 6B). IGF-2 transcript levels were significantly higher in the 13-dpf embryos compared to time 0 zygotes. Between 13- and 21-dpf, there was a significant decrease in IGF-2 mRNA levels; thereafter, IGF-2 mRNA levels increased over time up to 65-dpf, before decreasing significantly at 89-dpf (Fig. 6B). IGF-2 mRNA levels were significantly lower in embryos reared from oocytes treated with the higher BPA treatment relative to the controls at all time points examined except at 89-dpf where no significant difference was observed among the three treatments. Overall, IGF-2 transcript levels were lower in the BPA groups at 13-, 44- and 65-dpf, with dose dependent effects evident at 13- and 44-dpf (Fig. 6B).

IGF-1Ra and IGF-1Rb mRNA levels showed a gradual increase during early development, reaching maximum levels at 65- and 89-dpf (Figs. 6C and 6D). BPA exposure significantly impacted IGF-1Ra transcript levels with significant down-regulation observed in embryos reared from oocytes exposed to the higher BPA treatment for all the time points except 13-dpf (Fig. 6C). A dose-dependent effect of BPA was observed at 44- and 89-dpf, with the lower BPA treatment significantly down-regulating IGF-1Ra mRNA levels compared to the control group (Fig. 6C). Similarly, IGF-1Rb mRNA levels were also affected in the BPA treatment groups at 21-, 44- and 89-dpf, with dose-dependent effects at 21- and 44-dpf; no effect of BPA treatment was observed at 13- and 89-dpf (Fig. 6D).

### Stress Performance

**Figure 7:**
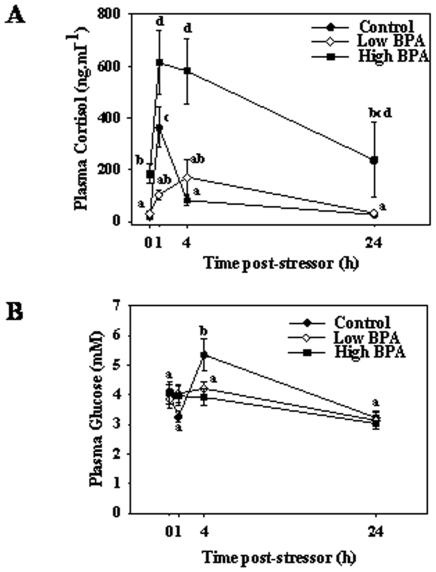
Impact of bisphenol A (BPA) on the organismal stress response. Effect of BPA exposure on stressor-induced plasma cortisol (A) and glucose concentrations (B) in juvenile rainbow trout (400-dpf). Plasma samples were collected at 0 (prior to stress), 1, 4 and 24 h after a handling disturbance. See Fig. 2 legend for details. All values represent mean ± SEM (n = 8 fish); time points with different letters are statistically significant (Two-way ANOVA; p<0.05).

Juvenile trout raised from oocytes exposed to high BPA had significantly higher basal plasma cortisol level compared to control group (Fig. 7A). No difference in basal cortisol levels was observed between control and low BPA groups. In the control group, as expected acute stressor significantly increased plasma cortisol levels at 1 h and the levels dropped significantly to pre-stress levels by 4 h and this was maintained over a 24 h period post-stressor exposure. In contrast to the control group, plasma cortisol response to stressor exposure was muted in the low BPA group and the levels were significantly lower than the control at 1 h post-stressor exposure but not at other time points (Fig. 7A). In the high BPA group, acute stressor significantly elevated plasma cortisol levels compared to the pre-stress levels as well as above those of the control and low BPA groups at 1, 4 and 24 h post-stressor exposure (Fig. 7A). Handling stressor significantly elevated plasma glucose levels at 4 h post-stressor compared to all the other time points in the control group. However, this stressor-induced plasma glucose elevation was not observed in fish reared from BPA exposed eggs (Fig. 7B).

## Discussion

Acute exposure of oocytes to BPA, mimicking accumulation of contaminants by maternal transfer, delayed development and reduced growth in rainbow trout. While no study has actually measured BPA levels in fish eggs, the dosage of this chemical in the present study (especially the low exposure group) reached levels similar to those reported recently for maternally transferred xenobiotics in feral fish eggs [12]. Although BPA was cleared from the embryos within 31 dpf, the growth suppression persisted even in juvenile fish, implicating long-term changes associated with chemical exposure at a critical period during early embryogenesis. This was accompanied by suppression of genes encoding for GH, IGFs and their receptors, underscoring the somatotropic axis as a key target for BPA-mediated developmental and growth disruptions.

The functional importance of the somatotropic axis during vertebrate development has been well demonstrated using gene knockouts and morpholino oligonucleotides (MO) in mice and zebrafish, respectively [16], [17], [18], [19]. The GH and IGFs transcript profiles associated with development growth trajectories in the present study are consistent with those seen in salmonids [20], [21]. However, to our knowledge, this is the first comprehensive study reporting the developmental expression of GH, IGFs and their receptors in a single study. The higher mRNA abundances of IGF-2 compared to IGF-1 prior to hatch supports a key role for this hormone in early embryogenesis, including embryonic cell cycle progression and differentiation [9], [20], [21]. In contrast, IGF-1 appears to play a major role in somatic growth in the post-hatched embryos [9] and this is in agreement with our observation of progressive increase in IGF-1 transcripts over time reaching maximum levels in hatched embryos (Fig. 5). The concurrent elevation in IGF-1R (both a and b isoforms) transcripts over time during development supports the concept that IGF signaling pathways play a key role during development [17], [18]. This may involve IGF-1 regulation of metabolic processes during early embryonic development as well as somatic growth in late stage embryos [9].

While IGF-1 expression is shown to be primarily regulated by GH in post-hatch embryonic stages [22], the role of GH in early stage embryos is still unclear. Several studies have hypothesized that GH acts as a mitogenic factor in early embryos [20], [23], [24], [25]; however, GH-MO did not impact early embryogenesis in zebrafish [19]. Consistent with this notion, although GH and GH-R transcripts were seen at all embryonic stages in the present study, significantly elevated levels were observed only in post-hatched embryos. Between the two isoforms, GH-2 and GH-2R transcript levels were comparatively higher than the GH-1 and GH-1R levels and is in agreement with the pattern reported previously in developing rainbow trout [20], [21]. Indeed, the increase in GH transcripts was reflected in elevated total embryo GH content and this coincided with increased embryonic growth. Also, the elevated GH content was associated with a significant upregulation in IGF-1 and IGF-2 transcript levels, supporting a role for somatotropic axis in growth regulation during later developmental stages in fish [24], [25].

This is the first study to indicate that the functioning of the somatotropic axis is impacted by exposure of eggs to xenobiotics prior to fertilization. Maternal transfer of contaminants from eggs to offspring has been shown to reduce growth and survival in fish [10], [11], [12], while the mechanisms involved are unclear. The present results demonstrate that BPA in eggs suppresses temporal mRNA abundances of IGFs and their receptors during embryogenesis, leading to delayed hatching and reduced growth. This involved BPA-mediated reduction in transcript levels of IGF-2 during early stages of embryogenesis (pre-organogenesis), while IGF-1 was affected in a dose-dependent manner post-hatching. In addition to the IGF pathway, there was also disruption of whole embryo GH content and GH-R transcript levels in the BPA groups. These changes were more pronounced after the beginning of exogenous feeding. The delayed hatching and the presence of yolk deposits in the BPA group suggests reduced nutrient absorption leading to growth defects during early life stages. Also in juveniles, the lower body mass corresponded with high plasma GH levels in the high BPA group, supporting a protein sparing role for GH similar to a fasting response in salmonid fishes [26], [27]. Taken together, these results suggest that BPA impact on the somatotropic axis functioning during early embryogenesis as a mechanism leading to developmental and growth impairment in juveniles.

Studies in mammalian models have shown that regulation of IGF system during embryogenesis is extremely sensitive to adverse environmental conditions, including maternal undernutrition, fetal hypoxia and infection [16]. It has also been hypothesized that any alteration to IGF signaling during early development results in permanent alterations to growth throughout life [28]. In fish, suppression of growth in adults was observed after chronic exposure (until 21-dpf) of embryonic rainbow trout to xenoestrogen [29]. This is consistent with our observation that acute BPA exposure of oocytes reduced growth even in adult trout (400-dpf) leading to the proposal that xenobiotic exposure during early embryogenesis may lead to long-term growth defects by disrupting the somatotropic axis. Although BPA was completely eliminated from the embryos within 35-dpf, the suppression of growth seen even in juveniles leads us to hypothesize that epigenetic modification of genes involved in the somatotropic axis function may be a mechanism resulting in long-term impact.

BPA is a weak xenoestrogen to fish and, therefore, some of the changes seen in growth may be related to the action of estrogen receptor signaling. For instance, there is great deal of information available on the interaction between hypothalamus-pituitary-gonadal axis and somatotropic axis in mammals, including regulation of postnatal growth, sexual dimorphism, metabolism, bone growth and nervous system development [30]. A similar level of understanding is lacking in teleost fishes; however, there is some evidence to suggest that there are potential sites of interaction between the two systems. This includes characterization of changes in ovarian GH-R binding sites throughout gametogenesis in rainbow trout and localization of IGF-1 and IGF-1R mRNA in rainbow trout testis [31], [32]. However, there is no direct evidence to suggest that E2 or xenoestrogens accumulation in oocytes disrupt growth in an ER-dependent fashion.

We were unable to investigate E2-mediated effects in the present study because exogenous E2 as well as ER antagonist (ICI182780), unlike BPA, were cleared from the oocytes within 24–48 h after exposure and did not result in VTG induction (data not shown). Previous studies have also shown that a one time exposure of embryos to E2 is ineffective because trout embryos were shown to metabolize exogenously-administered estrogens and thus not feminized by short-term estrogen exposure [33], [34]. We also did not observe any sex differences, as revealed by gonadal histology, between the treated and control groups (data not shown), supporting that feminizing effects of xenoestrogens are dependent on the mode and time of exposure [35]. However, the higher VTG mRNA abundances in embryos and juveniles of BPA exposed oocytes suggest activation of the ER signaling pathway. This would lead to a shift in energy utilization from somatic growth to vitellogenesis following BPA exposure. Previous studies have shown similar shifts in energy metabolism in males chronically exposed to E2 suggesting metabolic reprogramming as yet another mechanism of action by xenoestrogens to suppress growth [36]. However, the mechanism leading to higher VTG transcript levels and VTG protein expression at 140-dpf in the BPA groups, despite the absence of this chemical in the embryos, remains to be determined. We hypothesize that BPA exposure mimicking egg accumulation and/or maternal transfer alters DNA methylation patterns during early embryogenesis leading to persistent changes in the expression of genes, including VTG and genes critical for somatotropic axis functioning.

In addition to growth suppression, a key finding from this study was the disturbed plasma cortisol and glucose profiles in response to stressor exposure in adults developed from BPA exposed oocytes. Specifically, the disturbed cortisol and glucose response to a standardized physical stressor in BPA-exposed oocytes suggest disruption of the hypothalamus-pituitary interrenal axis functioning in trout. Indeed several studies showed that the cortisol stress axis is a target for xenobiotic impact leading to abnormal cortisol secretion in fish [37]. This could involve either changes to the steroid biosynthetic capacity, including disruption of genes encoding key proteins involved in corticosteroidogenesis [13] and/or changes to glucocorticoid receptor signaling, which is involved in the negative feedback regulation of plasma cortisol levels, as well as liver glucose regulation [38], [39]. The differing plasma cortisol response in the two BPA groups to a standardized stressor exposure is intriguing and warrants further study. The absence of a glucose response to stress in the BPA group also implicates a metabolic dysfunction as this metabolite is a key fuel for stress adaptation that is regulated by cortisol stimulation [40]. Overall, oocyte exposure to BPA leads to growth defects as well as long-term disturbances in the evolutionarily conserved cortisol and glucose response that is critical for acute stress adaptation in vertebrates.

In conclusion, maternal transfer of BPA and its accumulation in eggs affect offspring development, including growth suppression and altered stress performance in juvenile fish. Our results suggest that disruption of the somatotropic axis function during early embryogenesis may be involved in the growth and performance defects in juveniles. We hypothesize that epigenetic modification of genes critical for somatotropic and stress axes functioning may be a mechanism leading to long term growth suppression and reduced stress performance. Whether these phenotypic changes are transgenerational awaits further study. From a risk assessment stand-point, it will be essential to establish the threshold BPA (or other xenobiotics) dosage in eggs that will lead to long term growth impairment and reproductive dysfunction.

## Materials and Methods

### Experimental Fish

Experiments were conducted at Alma Aquaculture Research Station, Alma, Ontario, Canada, in accordance with the Animal Care and Use Committee of the University of Guelph, Ontario.

Pooled oocytes from six female rainbow trout (3+ year class brood stock) were used for all treatments. Approximately 500 oocytes were collected from each fish, and pooled together. The ovarian fluid from each fish was separated from the oocytes and stored prior to the experimental treatment. Pooled milt from six male fish (3+ year class) was used to fertilize all the eggs.

### Bisphenol A exposure

The pooled oocytes were distributed among three treatment groups, and duplicate groups were immersed in ovarian fluid containing either ethanol alone (0.01%; control group) or BPA-supplemented ovarian fluid containing final concentration of 30 and 100 µg.ml^−1^ for 3 h at 4°C. We used ovarian fluid as the medium in which to incubate the oocytes, because this is the fluid in which oocytes are retained post-ovulation in the peritoneal cavity for up to several days prior to release [41]. Previous studies have shown that thyroid hormone and even large molecules such as horseradish peroxidase can enter through the pore channels in the oocyte [42]. During the exposure period, the containers were gently shaken intermittently to ensure uniform exposure of all the oocytes. At the end of the treatment period, ovarian fluid with BPA was replaced with fresh ovarian fluid and oocytes were fertilized by the addition of approximately 1–2 ml of milt. One minute later, fertilization was stopped by the addition of water. Water hardened eggs were rinsed with fresh water several times before incubation. The fertilized eggs were incubated at 8°C in Heath chamber with a water flow rate of 10 liters/minute. Samples of embryos and juvenile fish were collected at time 0 (zygotes, immediately after fertilization and water hardening), 13- (organogenesis), 21- (eyed egg stage), 44- (hatching), 65- (first feeding) and 89- days post-fertilization (dpf) for transcript analyses. Body mass measurements for embryos were carried out every two weeks after the first feeding (65 dpf). Fish were maintained in continuous running water at 8°C and 12L: 12D photoperiod throughout the experimental period (until 400-dpf) and fed every hour using mechanical feeders. Sampling protocol involved euthanizing the embryos and juveniles in MS222 (Sigma) buffered with sodium bicarbonate and flash freezing them on dry ice. Samples were stored at −80°C until further analysis.

### Stress performance

Groups of 80 fish (400-dpf) each from different treatments (one sham and two BPA treated eggs) were distributed equally into 4 tanks (20 fish/200 L) and acclimated for one month prior to the experiment exactly as mentioned above. During this period, all groups (4 tanks per treatment ×3 treatments) were fed *ad libitum* with commercial feed every hour (5 point feed, Martin mills Inc, Elmira, Ontario). The stress protocol involved a handling disturbance that was described previously [13]. Briefly, at the beginning of the experiment, 8 fish from each treatment (2 fish from each tank) were sampled quickly and euthanized with an overdose of 2-phenoxyethanol (1∶1000) and these were the unstressed (0 h) control fish. The remaining fish were subjected to a handling disturbance for 5 min and were allowed to recover and sampled at 1, 4 and 24 h post-stressor exposure. Sampling consisted of quickly netting all fish from each tank and euthanizing the fish with a lethal dose of 2-phenoxyethanol (1∶1000). Fish were bled by caudal puncture into heparinized tubes and the plasma, collected after centrifugation (6000×g for 10 min), was stored frozen at −70°C for later determination of plasma cortisol and glucose levels.

### BPA levels in eggs

Tissue extraction and quantification of BPA analysis was carried out following the procedure described by Pedersen and Lindholst [43] with minor modifications. Briefly, fertilized eggs and developing embryos (pooled samples of 5 frozen individuals) were pulverized using mortar and pestle on dry ice and dissolved in a 20 ml mixture of dichloromethane: methanol (2∶1). The mixture was filtered through a Whatmann filter paper to remove any tissue debris, and 0.9% KCl solution was added to the final volume and centrifuged for 15 min at 1000×g. After centrifugation, the dichloromethane phase (organic) was removed and evaporated to dryness. The organic phase was redissolved in 1 ml of a mixture of methanol∶hexane (1∶20) and applied to a Sep-Pak NH2 500 mg cartridge pre-conditioned with 5 ml of methanol and 5 ml of methanol∶hexane (1∶20). The extraction cartridges were subsequently washed with 7.5 ml of hexane, dried for 3 min and eluted in 4 ml of methanol. After evaporation to dryness, each sample was re-dissolved in 300 µl methanol and used for BPA analysis.

Quantification of BPA was carried out using LC-MS/MS Method. Agilent 1200 was used for LC and Applied biosystems MDS Sciex API 3200 Qtrap was used for MS analysis. Chromatography was performed using Agilent Eclipse XDB-C18 (5 µm, 4.6×150 mm) column following established protocols. Calibration curve for BPA was established using BPA-d16 as a standard. The ratios of the peak areas of standard and the samples were calculated using Agilent chemstation software. The detection limit was determined at a signal to noise ratio >3 and the limit of quantification for BPA in the embryos were 75–80 ng.g^−1^ wet weight. The values were calculated and expressed as ng.embryo^−1^.

### Cortisol, growth hormone and glucose levels

Plasma cortisol levels were determined by radioimmunoassay following established protocol [13]. Growth hormone (GH) content in embryo and juvenile, as well as plasma GH levels were determined by enzyme linked immunosorbent assay (ELISA) following established protocol [44]. GH was extracted from embryo and juvenile samples (pools of 5 embryos or juveniles) prior to ELISA as described previously [45]. Plasma glucose levels were measured spectrophotometrically using a commercially available kit (modified Trinder method; Raichem, San Diego, CA).

### Quantitative real-time PCR (qPCR)

#### RNA isolation, cDNA synthesis and construction of plasmid stocks

Total RNA isolation from individual embryos and larvae was carried out using Qiagen RNeasy isolation kit (Qiagen, Ontario), and the RNA was quantified spectrophotometrically at 260 nm using Nanodrop. RNA was DNase treated to avoid genomic contamination, following manufacturer's instructions. RNA quality was determined by running 1 µg of total RNA on ethidium bromide stained 1% agarose gel electrophoresis. The first strand cDNA was synthesized from 1 µg of total RNA using First Strand cDNA synthesis kit (MBI Fermentas). Briefly, total RNA was heat denatured (70°C) and cooled on ice. The sample was used in a 20 µl reverse transcriptase reaction using 0.5 µg of oligo d(T) primers and 1 mM each of dNTP, 20 U ribonuclease inhibitors, and 40 U M-MuLV reverse transcriptase. The reaction was incubated at 37°C for 1 h and stopped by heating at 70°C for 10 min.

Primers for qPCR were designed based on the deduced rainbow trout cDNA sequences using Primer 3 software (see Table 1). PCR was performed to amplify the predicted products under the following conditions: initial denaturation for 3 min at 94°C, 40 cycles of 30 s at 94°C, 30 s at 60°C and 30 s at 72°C, followed by elongation for 7 min at 72°C. The resultant PCR products were subjected to 1.2% agarose gel electrophoresis and a single amplicon was detected. The PCR product was gel extracted, ligated into a pGEM-Teasy cloning vector (Promega,Valencia, CA, USA) and cloned into DH5α *Escherichia coli* cells. After LB-ampicillin selection, transformed cells were cultured and plasmids isolated using plasmid preparation kit (Sigma, ON). The isolated plasmid was sequenced at York University Molecular Core facility to confirm the presence of the amplicon, prior to its use for generating qPCR standard curves.

**Table 1:** Oligonucleotide primers of the genes used in quantitative real-time PCR along with their NCBI accession numbers, annealing temperature (Tm) and the size of the amplicon.

Gene name	Accession number	Forward Primer Sequence (5′-3′)	Reverse Primer Sequence (5′-3′)	Annealing Temperature (Tm; °C)	Amplicon size (base pairs; bp)
eF1α	AF498320.1	cattgacaagagaaccattga	ccttcagcttgtccagcac	56	95
IGF-1	EF450071	tggacacgctgcagtttgtgtgt	cactcgtccacaataccacggtt	68	120
IGF-2	EF450072	cggcagaaacgctatgtgga	tgctggttggcctactgaaa	58	79
IGFRIa	AF062499	agagatagacgacgcctccta	caccaaatagatccctacgt	58	104
IGFRIb	AF062500	cctaaatctgtaggagacctggag	gttagccacgccaaatagatcc	58	139
GH-1	AF005923	ttcaagaaggacatgcacaaggtc	ctccagcccacgtctacaga	66	97
GH-2	DQ294400	cccacgtttacagagtgcagttg	gcttcaagaaggacatgcataaggtt	66	93
GH-R1	AY861675.1	tgaacgtttttggttgtggtcta	cgctcgtctcggctgaag	60	61
GH-R2	AY751531.1	catggcaacttcccacattct	gctcctgcgacacaactgttag	60	65
VTG	AJ011695	caagatcgatcggaagggta	ccacaggtctgtcccttcat	60	121

#### Standard curve

Plasmids with target sequence inserts were used for establishing standard curves. Standard curves were generated using a serial dilution of the plasmids to attain varying copy number of insert sequences (10^8^–10^1^ copies). Each standard reaction mix contained 1 µl of cDNA, 4 pM of each primer and SYBR green super mix (50 U.ml^−1^ of iTaq DNA polymerase, 40 mM of Tris-HCl (pH 8.4), 100 mM of KCl, 6 mM of MgCl_2_, 0.4 mM of each dNTP component (dATP, dGTP, dCTP and dTTP), SYB Green I, 20 µM of flouresein, and stabilizers) in a total volume of 25 µl. PCR was performed using iCycler iQ™ (BioRad) under the following conditions: 2 min at 94°C followed by 40 cycles of 15 s at 95°C and 30 s at respective annealing temperature. PCR products were subjected to melt curve analysis to confirm the presence of a single amplicon. Control reactions were conducted with no cDNA template and with RNA to determine the level of background or genomic contamination. Master mixes, to reduce pipetting errors, were prepared at every stage for triplicate reactions (3×25 µl) for each standard. Background subtracted threshold cycles (C_T_) values were plotted against log of standard copy numbers to obtain standard curves. The PCR efficiency (E) was determined using the formula, E =  [10^−(1/slope)^*100] and it ranged from 96–100%.

#### Quantification

One µl of cDNA sample was used as a template for every 25 µl reaction. For every test sample, qPCR was performed for both the gene of interest and the housekeeping gene (elongation factor 1alpha: eF1a). The reaction components, PCR conditions and melt curve analysis were exactly same as the previous section. Background subtracted threshold cycle (C_T_) values were used to determine the absolute quantity of the mRNA based on the standard curve. Elongation factor-1a (eF1a) mRNA levels showed little change with age or experimental treatment and these were used for the normalization of transcript abundance.

### SDS-PAGE and Western blotting for vitellogenin (VTG)

Protein concentrations in the liver were determined using the bicinchoninic acid method with bovine serum albumin as the standard. The procedure for SDS-PAGE and western blotting were according to established protocols [40]. Briefly, samples (40 µg protein/sample) were separated on 6% polyacrylamide gels using a discontinuous buffer system [46]. The proteins were transferred onto a nitrocellulose membrane (20 V for 20 min) with a semidry transfer unit (BioRad) using transfer buffer (25 mM Tris pH 8.3, 192 mM glycine, and 20% (v/v) methanol). The membrane was blocked with 5% skimmed milk in TBS-t (20 mM Tris pH 7.5, 300 mM NaCl and 0.1% (v/v) Tween 20 with 0.02% sodium azide) for 60 min. The rabbit polyclonal anti-trout VTG primary antibody (1∶5000; generated in-house) and alkaline phosphatase-conjugated goat anti-rabbit secondary antibody (1∶1000; BioRad) were diluted in the blocking solution. The membranes were incubated in primary antibody for 60 min at room temperature, washed with TBS-t (2×5 min), incubated with secondary antibody for 60 min, washed with TBS-t (2×5 min), and finally washed with TBS (1×15 min). Visualization of bands was carried out with NBT (0.033% w/v) and BCIP (0.017% w/v) and the molecular mass was confirmed using prestained low molecular weight marker (BioRad). Quantification of bands was carried out with Chemi imager™ using the AlphaEase software (Alpha Innotech, CA).

### Statistical analysis

All statistical analyses were performed with SPSS version 14.0.1 (SPSS Inc., Chicago, IL, USA) and data are shown as mean ± standard error of mean (SEM). The data were log-transformed, wherever necessary, for homogeneity of variance, but non-transformed values are shown in the figures. The tests used were either one-way or two-way analysis of variance (ANOVA) and a Bonferonni *post-hoc* test was used to determine statistical significance between groups. A probability level of p≤0.05 was considered significant.
